# Biological Activity and Binding Site Characteristics of the PA1b Entomotoxin on Insects from Different Orders

**DOI:** 10.1673/031.007.1201

**Published:** 2007-03-08

**Authors:** Frédéric Gressent, Gabrielle Duport, Isabelle Rahioui, Yannick Pauchet, Patrice Bolland, Olivier Specty, Yvan Rahbe

**Affiliations:** ^1^ UMR203 Biologie Fonctionnelle Insectes et Interactions, IFR41, INRA, INSA-Lyon, F-69021 Villeurbanne, France.; ^2^ INRA 1112 - UMR ROSE, 400 Route des Chappes, 06903 Sophia Antipolis Cedex, France

**Keywords:** PA1b, binding site, toxin, knottin, cystine-knot peptide

## Abstract

The aim of this work was to investigate both the biological activity of an entomotoxin, the pea albumin 1b (PA1b), and the presence or absence of its binding site within an array of insect species. The data obtained showed that insect sensitivity was not related to its taxonomic position. Moreover, PA1b was not toxic to several tested microorganisms. However, the binding site was found to be conserved among very different insects, displaying similar thermodynamic constants regardless of the *in vivo* species sensitivity. The binding site alone was, therefore, not sufficient for toxicity. One exception was the pea weevil, *Bruchus pisorum,* which was the only tested species without any detectable binding activity. These findings indicate that the binding site probably has an important endogenous function in insects and that adaptation to pea seeds resulted in the elimination of the toxin binding activity in two independent insect lineages. Other mechanisms are likely to interact with the toxin effects, although they are still largely unknown, but there is no evidence of any specific degradation of PA1b in the midgut of insects insensitive to the toxin, such as *Drosophila melanogaster or Mamestra brassicae.*

## Introduction

The cereal weevils, *Sitophilus oryzae, Sitophilus granarius* and *Sitophilus zeamais,* are major pests of stored grains. Currently, the use of chemical insecticides is the main solution to prevent the damage caused by stored product pests, inducing ecotoxicity problems and the occurrence of resistance within insect populations. Ideally, plant protection could take advantage of the genetic resources residing in crop plants to create resistant varieties, and develop the use of biological peptide toxins to build up transgenic plants tolerant to different insect species. The *Bacillus thuringiensis* toxins (Schnepf et al. 1998), proteins belonging to the lectin family (Peumans and Vandamme 1995), or enzyme inhibitors (Gatehouse and Gatehouse 1998) are currently in use, or being tested, but to date these molecules are not adapted for grain protection against weevils. A vitamin scavenger has been shown to protect maize against the larval stages of *S. granarius* (Kramer et al. 2000), but it may induce intestinal or regulatory problems if used in human nutrition.

PA1b (for Pea Albumin 1b) is a plant peptide purified and sequenced from seeds of the pea, *Pisum sativum,* (Higgins et al 1986) that is lethal for certain insects, such as the cereal weevils or the pea aphid *Acyrthosiphon pisum* (Delobel et al. 1998), although the fruit fly *Drosophila melanogaster* has been found to be insensitive. PA1b consists of 37 amino acids (-ASCNGVCSPFEMPPCGTSACRCIPVGLVIGYCR NPSG-), with six cysteines involved in three disulfide bonds that give the toxin its high stability. Moreover, PA1b might belong to a multigenic family since at least five isoforms of the peptide exist in a single pea genotype, and it seems to be widespread in legumes (Louis et al 2004). It was recently established, by NMR studies and molecular modeling, that PA1b belongs to the cystine-knot family (Jouvencal et al. 2003), which has now been confirmed from a variety of sources. Cystine-knot peptides show very diverse biological activities (for review see Craik et al. 2001). PA1b was the first entomotoxic cystine-knot peptide identified, recently joined by a member of the cyclic peptide family kalata B (Jennings et al. 2001).

*S. oryzae* strains resistant to PA1b exist naturally. Screening of up to 90 *Sitophilus* spp. strains for susceptibility to PA1b has revealed that three strains, all belonging to the *S. oryzae* species, contained individuals able to feed on pea seeds (Grenier et al. 1997). These strains were also fully resistant to the purified toxin. A genetic analysis of the resistance demonstrated that this trait was driven by a single recessive autosomal gene (Grenier et al. 1997). This result suggests that a single weevil gene product could be responsible for the susceptibility or resistance towards the toxin.

On this basis, the search for the molecular mechanism of action of the toxin focused on the hypothesis of a receptor on the target insect. Biochemical studies showed the presence of a proteinaceous high affinity binding site for PA1b on *Sitophilus* membrane extract. This binding site was present on all susceptible *Sitophilus* species and strains, but was not detectable on extracts from the four *S. oryzae* resistant strains (the three natural strains, plus one strains obtained in our laboratory by back-crossing a resistant into a sensitive genotype) (Gressent et al. 2003). Thus, the binding protein was probably involved in the toxicity process and the resistance of strains the *S. oryzae* species was due to the absence or the mutation of the target molecule. However, speices insensitive to the toxin, such as the fruit fly, displayed a binding site for PA1b similar to that found on *Sitophilus,* indicating that other(s) mechanism(s) of resistance occur among insect species.

Therefore, the aim of the present work was to investigate the biological activity spectrum of PA1b among insect families, as well as on some fungal and bacterial species. The presence and the characterization of the PA1b binding site was also investigated in the tested insects.

## Materials and Methods

### Biological material and toxicity assays

Cereal weevils *(Sitophilus oryzae, S. zeamais* and *S. granarius,* Coleoptera) were reared for more than 10 years in our laboratory on wheat seeds at 27-5°C 70% RH. Resistant strains were reared on pea seeds. Tests and survival analysis were performed on adults, as fully described in Louis et al (2004).

Growth and toxicity assays were carried out on the aphids *Aphis gossypii, Myzus persicae* and *Acyrthosiphon pisum,* Hemiptera, according to Rahbé and Febvay (1993). Larvae of the fruit fly *Drosophila melanogaster,* Diptera, were provided by Dr. Allemand (UMR 5558 CNRS-UCBL, Lyon, France).

The red flour beetle *Tribolium castaneum* (Coleoptera), was provided by François Bonneton (Centre de Génétique Moléculaire Cellulaire, Lyon, France). Tests were performed on adults by mixing the toxin into the diet (wheat flour 95%, yeast extract 5%).

*Trichogramma pretiosium.* T191 (Hymenoptera) were grown using the artificial diet described in Grenier et al. (1998). The toxin was incorporated into the diet, and the development of insects was monitored from eggs to adults.

The ladybeetle *Harmonia axyridis* (Coleoptera) was grown on a pork liver-based artificial diet (Specty et al., unpublished results), and tests were performed using a diet supplemented with the toxin. The development was monitored from L3 up to the pupal stage.

The mosquito bioassay was performed on larvae of *Culex pipiens* (Diptera) in water. The toxin was added directly to the water, and the mortality of L3 larvae was recorded daily.

The cabbage moth *Mamestra brassicae* (Lepidoptera) was grown on the artificial medium described by Poitout and Bues (1974). PA1b was added to this media and the mortality of larvae (from L1 to pupa) was monitored daily.

All toxicity assays were carried out using a mixture of PA1b isoforms (98% purity by HPLC). All tests were performed on at least 30 insects for assays and for control test (without toxin), except for the three aphid species (50 insects) and for Trichogramma (more than 300 eggs by assays). When adults were used, the insects were synchronized at the imaginal molt. The statistical analyses were performed using the JMP software (SAS, Grégy-sur-Yerres FRA).

### Insect extracts

The isolation of membrane proteins for binding studies was performed as described by Gressent et al. (2003). For PA1b degradation tests, insect extracts were prepared as follows, 1 g of insects were ground with a mortar and pestle in liquid nitrogen. The resulting powder was resuspended in 2 ml of extraction buffer (20 mM Tris-HCl pH 8; 0.25 M sucrose; 2 mM MgCl_2_). The slurry was centrifuged for 2 min at 1000 *g,* and the supernatant was stored at -2O°C until required. All the operations were performed at 4°C.

### Purification of the toxin

Purified toxin isoform (PA1b-3741), with a molecular mass of 3741 Da by mass spectrometry, and a pea albumin extract (SRA1) were provided by J. Gueguen and E. Ferrasson (Laboratoire de Biochimie et Technologie des Protéines, Nantes, France). From SRA1, a mixture of PA1b isoforms was obtained using to the methods of Gressent et al. (2003).

### Binding assays

The peptidic toxin PA1b-3471 isoform was labelled with I^125^ to a specific radioactivity of about 900 Ci.mmor^-1^, and then used for binding studies with membrane extracts, according to Gressent et al. (2003).

### PA1b degradation assays

PA1b degradation assays were performed using 10 µg of peptide (PA1b isoform mixture) in a final volume of 100 µl buffer. Assays with commercially (SIGMA, www.sigmaaldrich.com) available enzymes were performed in Glycine-NaOH buffer (20 mM pH 9) at 3O°C for proteinase K (E.C. 3.4.21.64), Tris-HCl buffer (20 mM pH 7.5) + EDTA 10 mM at 37°C for pronase E type XIV, MES-NaOH buffer (20 mM pH 6.2) at 25°C for papain (E.C. 3.4.22.2), Tris-HCl buffer (20 mM pH 7.8) at 25°C for chymotrypsin (E.C. 3.4.21.1), and Tris-HCl buffer (20 mM pH 7.6) at 25°C for trypsin (E.C. 3.4.21.4). Assays were done with 10 µg of each enzyme, and control assays were performed with 10 µg of enzymes denaturated by 10 min boiling. Assays with insect extracts were carried out in 20 mM buffers from pH 3 to 9 (Glycine-HCl, pH 3 ; sodium acetate pH 4-5 ; MES-NaOH pH 6 ; Tris-HCl pH 7-8 ; Glycine NaOH pH 9) at 3O°C. In test assays 200 µg of insect extract proteins were added (400 µg when using the *M. brassicae* gut content), and controls were performed with similar amounts of proteins denaturated by 10 min boiling. Each point was the mean of triplicates.

For all assays, after a one night incubation, 150 µl of methanol were added to the mixture and incubated for 60 min at 4°C. After centrifugation for 10 min at 10,000 g 200 µl of the supernatant was injected on a reverse phase C18 HPLC column eluted at 1 ml.min^-1^ with a gradient of water; TFA 0.1% / acetonitrile ; TFA 0.1% (80/20 for 2 minutes, then 40/60 for 20 min). PA1b peptide isoforms were detected by their absorbance at 210 nm, quantified by the measurement of the peak area and with reference to known quantities of pure peptide isoform used as standards.

### Protein determination

Protein content was measured by the bicinchoninic acid procedure developed by Pierce (www.piercenet.com) with bovine serum albumin as the reference.

**Figure 1.**
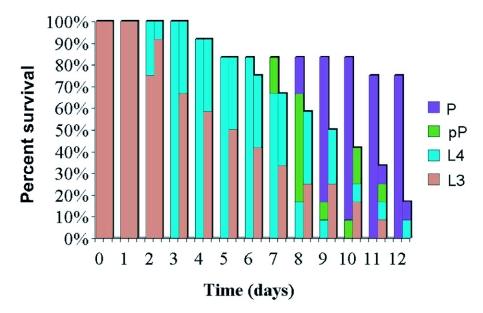
Effects of PA1b on the lady beetle *H. axyridis.* Tests were done with artificial diet supplemented with the toxin (250 µg/g), and the development of the insects was monitored daily. First bar, control insects; second bar, tested insects. P, Pupae; pP, Pre-pupae; L_4_,4^th^ instar larvae; L_3_, 3^rd^ instar larvae.

## Results

### Biological assays of PA1b toxicity on different insect species

Dose-response curves, performed on different strains of the genus *Sitophilus,* showed that LD_50_ values at day 7 are for *S. oryzae* (Bénin, Bouriz and WAA42) 446 [251–1584], 501 [281–984] and 489 [290–1318] µg/g respectively, with confidence intervals shown in brackets. The three LD_50_ show no statistical difference (α= 0.05). The *S. zeamais* LS strain had a LC_50_ of 234 [125–630] µg/g. This value is statistically different from the three *S. oryzae* strains LD_50_ (α= 0.05). For *S. granarius* Brayard strain the highest tested dose (1000 µg/g ; 27 % mortality) did not allow the calculation of a precise LD_50_. This value has been estimated by extrapolation to ca. 3500 µg/g, without confidence interval.

The ladybeetle *H. axyridis* was tested with a relatively low concentration of PA1b (250 µg/g of food), and the test was started on L3 larvae. When compared to the control insects (diet without PA1b, see Figure 1), differences appeared after three days of assay: all control insects reached the L4 stage, while only 30% of the intoxicated insects did so. Afterwards the difference between the two groups increased, control insects growing more rapidly and mortality occurring in the tested insects. By day 12, 75% of the control insects had survived and reached the pupal stage. In contrast only 15% of the tested insects had survived, and only half of these survivors reached the pupal stage.

We next tested the effect of PA1b on adults of *T. castaneum.* In contrast to other Coleoptera, no effects were detectable in *T. castaneum,* and all insects survived even after feeding for 15 days on a high dose of PA1b (1000 µg per g of food).

*Ephestia kuehniella* was previously found to be sensitive to the toxin (Delobel et al 1998). In contrast, 1000 µg PA1b per g of diet had no effect on the cabbage moth *Mamestra brassicae,* either on the mortality or on the development and ecdysis of the insects (data not shown).

*D. melanogater* has previously been shown to be insensitive to the toxin (Delobel et al.1998). Toxicity tests performed on another diptera, *Culex pipiens,* showed that L3 larvae of this mosquito were rapidly killed by moderate concentrations of PA1b (250 µg.ml^-1^ of water): survival was 100% after one day, and 0% after two days.

Wasps of the genus *Trichogramma* are often used for biological control of certain pest insects. Adding PA1b to an artificial diet allowed normal growth of the species *T. presiosum,* leading to a complete development from eggs to adults, with no difference between developmental times could be observed compared to control insects reared on a PA1b-free diet.

**Figure 2.**
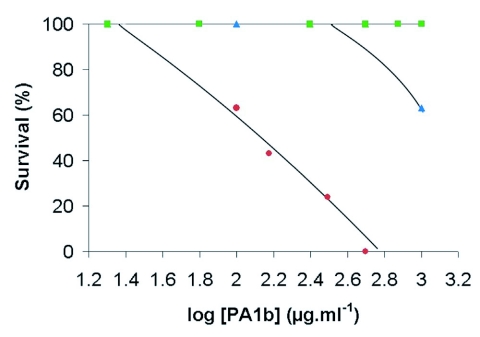
Effects of PA1b on three aphid species. Tests were carried out with artificial diet supplemented with the toxin at increasing concentrations, and mortality of the insects was monitored daily (relative larval survival is used in the graph). Circle : *A. pisum* ; Square : *M. persicae;* Triangle : *A. gossypii.*

Three aphid species were tested for their sensitivity to PA1b. The results presented in Figure 2 show that *A. pisum's* survival decreased rapidly with PA1b concentration. After six days feeding on PA1b diets, observed mortality was 57% at 150 µg.ml^-1^, and was total at 500 µgml^-1^. In contrast, no mortality was observed for *M. persicae* at doses up to 1000 µg.ml^-1^ while a slight effect was observed at this high concentration only for *A. gossypii* (37 % lethality).

**Table 1.**
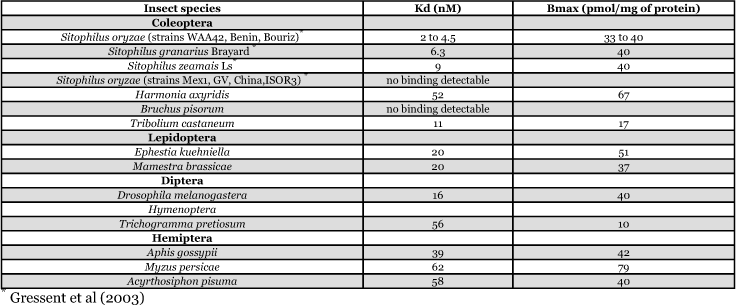
Affinity (Kd) and relative abundance (Bmax) of the PA1b binding protein on membrane extracts from different insect species and strains.

### Biological tests on fungi and bacteria

A possible antimicrobial and antifungal activity of PA1b was next tested on four bacterial and three fungal species. The experiments were done using an increasing toxin concentration, up to 64 µg-ml^-1^. At this concentration no effect was detected, either on bacterial survival (*P. aeruginosa, S. aureus, E. faecium* and *E. coli),* or on fungal growth (*C. albicans, C. glabrata* and *A. famigatus),* 24 and 48 hours after the addition of the toxin.

**Figure 3.**
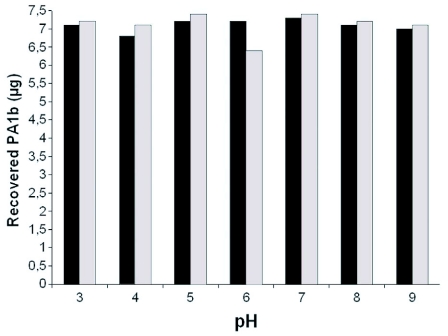
Effects of *A. gossypii* protein extracts on PA1b. PA1b was incubated with 200 µg of *A. gossypii* proteins in a solution buffered from 3 to 9. Degradation of PA1b was monitored after incubation by reverse-phase HPLC. Control assays were done using heat-denaturated insect protein.

### Characteristics of the PA1b binding site

Previous studies have shown the existence of a high-affinity binding site on *Sitophilus* membrane extracts. The absence of this protein in membranes from resistant weevil strains leads to the conclusion that the binding protein plays a key role in PA1b toxicity. Therefore, the presence of binding was investigated in membrane extracts of various tested insect species, and its affinity and relative abundance were calculated.

Data obtained from competition experiments, using the purified 3741 kDa isoform as competitor, are shown in Table 1. PA1b binding activity was present in all insect species, except for the resistant weevil strains and the pea specific pest *Bruchus pisorum,* regardless of the sensitivity or insensitivity of these insects to PA1b toxicity, as evaluated from our insect bioassays. Binding affinity for PA1b was found to be slightly different in the tested species, ranging from 2 nM *(Sitophilus oryzae)* to a maximum of 62 nM *(Myzus persicae).* The relative abundance of the binding sites was also slightly different in this group of species, varying from 10 pmol/mg of protein for *T. pretiosum* to a maximum of 79 pmol/mg of protein for *M. persicae.*

**Table 2.**
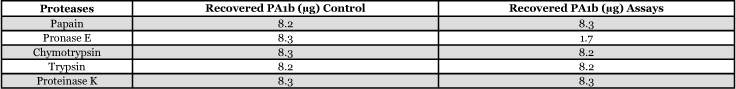
Degradation assays of PA1b by proteases.

### PA1b degradation assays

Since the results from binding experiments clearly showed that the presence of the binding site and PA1b toxicity were not correlated (except for *B. pisorum* that thrives on pea seeds), the hypothesis of a differential degradation of the toxin in the insect gut was tested by examining the ability of different proteases to hydrolyse the PA1b peptide.

The data presented in Table 2 demonstrate that for trypsin, chymotrypsin, proteinase K and papain, the amount of PA1b recovered after degradation tests (about 8.3 µg) was essentially the same as quantities obtained after control tests with denatured proteases. These proteases were therefore unable to hydrolyse the toxin, even under the optimal conditions for these enzymes. However, after treatment with the pronase E enzyme, only 20% of the control PA1b was recovered; pronase E was the only enzyme able to hydrolyse the peptide toxin.

To assess the ability of insect extracts to hydrolyse the toxin, experiments were done using extracts of total insect proteins. Three species that were insensitive to the toxin were chosen from three different orders: *M. brassicae* (Lepidoptera, L3), *D. melanogaster* (Diptera, L3) and *A. gossypii* (Hemiptera, L4). The results presented in Figure 3 show that for a pH ranging from 3 to 9, the recovered PA1b after incubation with 200 µg of A. *gossypii* proteins was very close to the control test, realized with the same amount of insect protein extract that had been denatured by boiling. The same experiments, using *M. brassicae* or *D. melanogaster* protein extracts assayed in the same pH range, led to similar results without any difference between control and test assays (data not shown).

We next used gut extracts from *M. brassicae* for degradation assays, and increased the protein concentration to 400 µg per assay. Even in these conditions, using a pH ranging from 3 to 9, no difference was found in the recovery of PA1b after incubation with native or heat inactivated gut proteins (data not shown). Thus, it seems clear from these results that insect proteases from insects insensitive to the toxin were not able to degrade the toxin.

## Discussion

The entomotoxic properties of the PA1b peptide could represent a valuable tool for cereal protection against weevils, and even more generally for plant protection against pest insects. In order to evaluate the potential usefulness of this toxin, and any possible extension of its use, our present work was aimed at addressing two important issues:

1) A more accurate determination of the host range of the toxin, on either pests or non-target or even useful insects. Published data only concern a few insect species from different orders (Gressent et al. 2003). The sensitivity of microorganisms to this class of legume seed albumins was also unknown. Identifying a correlation between insect taxonomy and both the lethal effects or the presence of the high-affinity binding site in target tissues was also one of the aims of this paper.
2) Understanding more precisely the mechanism by which insensitive insects might overcome the toxin's effects. The PA1b toxicological model in the *Sitophilus* species clearly involves a binding site, but data on other species were still too scarce to provide a global idea of the importance of this binding step in the advent of the toxic syndrome.

### Absence of taxonomical correlation

Toxicity assays performed on different insect species showed that there was no correlation between the insect family and toxin effects. Even among the aphid species tested, strong differences could be observed, with effects of the toxin ranging from readily toxic *(A. pisum)* to almost non-toxic (*M.* *persicae*)*.* Such species-specificity to toxins is not uncommon in aphids with, in general, the specialist species showing the strongest susceptibility *(A. pisum)* while more generalist species, such as the peach-potato aphid (*M.* *persicae*) or the cotton-melon aphid *(A. gossypii)* that tend to be more resistant to other peptide toxins (Rahbé et al. 2003a; Rahbé et al. 2003b). Even within a single tribe of aphids, the Macrosiphini, the very weak activity of one species, *Myzus persicae,* does not preclude the potency of PA1b on another species, *Acyrthosiphon pisum.* This situation is reminiscent of that encountered for the peptide inhibitor oryzacystatin, being weakly inhibitory on most tested aphid species, including *A. pisum* and *M.* *persicae* (Rahbé et al. 2003b), but strongly lethal at a similar dose to the potato aphid *Macrosiphum euphorbiae,* also belonging to the same Macrospiphini tribe (Azzouz et al. 2005). *T. castaneum* was found to be insensitive. Hou et al (2004) show a slight effect of pea flour (containing PA1b) on *T. castaneum* population. This difference could be due to difference in the purity of the toxin (pea flour/pure isoforms), or most probably to variations in experimental method and / or doses. The absence of taxonomical correlation to a biological effect of PA1b, at different taxonomical scales, is therefore well documented for peptide toxins, and contrasts with the proximal molecular interaction which may rely in many cases, as occurs for PA1b and *Sitophilus,* on a simple genetic determinism.

### The A1b binding site is generally present in insect tissues

One striking result of the present study was the wide occurrence of a specific binding site in tissues from very different insects. In almost all tested species, a specific binding was detected with low variability. Affinity constants ranged from 2 to 60 nM, indicating a high conservation of the target binding site across the analysed taxa. Similarly the relative abundance of this activity was not very variable in the screened tissues. This finding is more unusual than the previous point discussed, as it does not seem to occur for interactions with well-characterized toxin / binding site couples, such as *B. thuringiensis (Bt)* toxins or protease inhibitors, for example. The general situation for *Bt* toxin specificity is that there is a good correlation between toxin binding and its biological outcome, either at the insect-species level (Hofmann et al. 1988) or at the insect-instar level (Rausell et al. 2000), although some more complex situations might occur with developmental factors (Gilliland et al. 2002).

### Binding site is not a sufficient condition for susceptibility

Stating that the binding activity is widely present, while the toxicity is not, directly implies that other factors strongly influence the toxin toxicity. Pre-binding effects, such as toxin stability in the insect digestive tract, have been commonly described, either for bacterial toxins (Keller et al. 1996) or enzyme inhibitors (Girard et al. 1998). Although the structural features of PA1b, such as its cystine-knot core (Jouvensal et al. 2003), are known to confer high thermal, chemical and proteolytic stability (Delobel et al. 1998) the latter point was investigated using commercial and crude insect endoproteases. PA1b was found to be insensitive to all purified enzymes tested, except for pronase, which is consistent with results obtained by Taylor et al 2004. Pronase is a microbial protease mixture preparation with low specificity (i.e. it is able to cut many amino-acid residues) compared to a protease like trypsin. Similarly, none of the midgut preparations used in this study were shown to alter PA1b, and midgut extracts from sensitive or resistant S. oryzae do not show any activities on PA1b (Delobel and Rahbé, personnal communication).

*A. gossypi* and *S. oryzae* gut proteases are mainly of the cysteine type (Deraison et al. 2004; Liang et al. 2004), but noctuids display serine type proteases for digestion (Hegedus et al. 2003). These results demonstrates that digestive degradation, a widespread mechanism of toxin inactivation, was not involved in the observed insensitivity.

### Diet effects exist, as do other factors

Insect diet could be one of the factors influencing sensitivity to PA1b. While *D. melagonaster* was shown to be very insensitive when reared on a commercial artificial diet, when larvae were grown on a fruit-based mixture PA1b was found to be significantly toxic, delaying larval development and increasing overall larval mortality at a dose similar to that used on other insects (Gressent, unpublished results). However, diet is not sufficient alone to explain insensitivity, as the three aphid species tested were assayed on the same artificial diet with very different results. We can thus suppose that a certain number of factors could interfere with the toxin activity, from diet to pre- or post- binding effects (e.g. mutation in a putative protein acting in the signal transduction).

### Pea seed insects lack the albumin binding site

Out of all the insect species, only one of them, *B. pisorum,* is a natural pea seed pest. *B. pisorum* is also the only species where the binding activity was totally undetectable. This was also true of some strains of *S. oryzae,* but the species itself could be regarded as possessing the binding site. Thus, this result seems to indicate that the suppression of PA1b binding activity is the best and most efficient way to overcome plant defense through PA1b accumulation, even though other mechanisms do exist in insects feeding on non-legume hosts.

PA1b toxic effects could not be anticipated, either from insect taxonomy or by the presence of the binding site. This result may be a problem in using PA1b for pest control as its effects on useful insects, for example, could not be anticipated. The only way to evaluate these effects is to perform a biological test. However, the absence of any binding activity should result in a resistant phenotype. The toxin showed no effect on microorganisms.

The binding site seems to be widely and well conserved among insect orders as it was found to display similar properties in very diverse species. This indicates that the target protein probably has an important and conserved function. Also, toxin binding needs to be eliminated to gain efficient adaptation to pea seed feeding. Whether this process is costly or not, and whether it involves the elimination or the modification of the target, is still not known. However, we may compare this conservation on the part of the insect to the conservation of the toxin as regards the plant, which has been demonstrated at both gene and peptide levels (Louis et al. 2004) within the *Papilionoidae* clade of the *Fabaceae.* Although a high variability exists at the plant species level, homologous genes and bioactivities were detected widely within a whole branch of the legume family, indicating a long lasting selective pressure for maintaining this peptide family and properties, which may be an indication of its use as a general insect toxin. This present set of observations reinforces interest in identification of the binding-site, as this molecular target could represent a new target for insecticides.
